# Study of knowledge, attitude and practice regarding patient education in hypertension among community pharmacists in China

**DOI:** 10.1186/s12913-022-08686-9

**Published:** 2022-10-28

**Authors:** Lei Chen, Yueyue Liu, Xiaoyu Xi

**Affiliations:** China Pharmaceutical University, No.639 Longmian Avenue, Jiangning District, Nanjing, Jiangsu Province, China

**Keywords:** community pharmacist, hypertension, patient education, knowledge, attitude and practice

## Abstract

**Background:**

In the prevention and treatment of hypertension, patient education is an important measure to improve the awareness rate and control rate of patients. The professional and geographical advantages of community pharmacists enable them to play an important role in the patient education in hypertension. The purpose of this study was to understand the situation of patient education in hypertension conducted in Chinese community pharmacies, and put forward measures according to the problems.

**Methods:**

A multi-stage competitive sampling by convenience was used to select community pharmacists working in community pharmacies in China for the study. Based on KAP theory, the first draft of the questionnaire was designed and the Delphi method was used to improve the questionnaire and a pre-study was conducted to test the reliability of the questionnaire. In January 2020, electronic questionnaires were distributed to 143 community pharmacists in Chinese community pharmacies. SPSS24 software was used for descriptive statistics and subgroup analysis of data.

**Results:**

One hundred and eight valid questionnaires were collected, and the efficiency rate was 75.5%. Most of the respondents were younger than 30 years old (98.1%), and had bachelor’s degree (95.4%). In terms of knowledge, only 15.7% considered themselves "very good" and even 10.2% considered themselves "very bad". Only 35%-55% of respondents answered correctly for patient education content that requires more specialized knowledge, such as treatment and medication. Respondents generally had a positive attitude on the effect of hypertension patient education, but slightly less recognition of their role in patient education. In terms of practice, programs related to patient education have been conducted to different degrees. More than 30% of the community pharmacists interviewed implemented them occasionally or never.

**Conclusions:**

Despite a positive attitude, most of the respondents did not have a high level of knowledge or practice. In China, more research evidence and new guidelines are needed to emphasize the importance and responsibilities of community pharmacists. Continuing education should be certificated at the national level and meet the various needs of community pharmacists. And salary incentives can be tried to motivate them.

## Background

Hypertension is the leading risk factor for cardiovascular diseases [1]. Hypertension management is essential for controlling disease advancement and promoting residents’ health. Patients’ better knowledge of hypertension can improve their compliance, and consequently, better compliance is conducive to controlling their blood pressure (BP) [2–4]. Therefore, as an important part of hypertension management, patient education is worth of research. Patient education refers to increasing patients’ knowledge of disease through education, which can give patients a deeper understanding of the characteristics of the disease, discipline patients’ behavior, enhance clinical outcomes, or more specifically improve patients’ medication compliance and BP control effectively [5, 6]. The official guidelines for hypertension management of various countries have emphasized the importance of patient education to different degrees [7–9]. For example, management of Arterial Hypertension in Adults issued by the French Society of Arterial Hypertension recommends that patients should conduct a consultation that specifically provides disease and treatment information before drug treatment [7]. Guidelines for Hypertension Education in China (GHEC) has directly clarified the content, methods and skills of patient education for medical staff [8]. The Canadian government has issued guidelines, stating that pharmacists should educate hyperation patients and their family members, which can significantly reduce systolic blood pressure (SBP) and diastolic blood pressure (DBP) [9].

Since there is still a gap between the awareness rate and control rate of hypertension patients in China and that of developed countries, focusing on the patient education conducted in community pharmacies is an important step in addressing the challenges brought by hypertension. According to the China Hypertension Survey, the crude prevalence rate of hypertension among residents aged over 18 from 2012 to 2015 was 27.9%, only 51.6% of the patients were aware of their health condition and 16.8% had their condition under control [10]. Although the awareness rate and control rate have been significantly increased, they remain lower than those in U.S. and Canadian, which are average of about 80% and 55%, respectively [11, 12]. In addition, there is lack of disease knowledge among Chinese hypertensive patients [13–15]. Patient education conducted by community pharmacists is an effective way to ensure the hypertension management and enhance the awareness rate and control rate of patients. Several studies from different countries have shown that interventions implemented by community pharmacists can improve blood pressure control, with the most frequently implemented ones including patient education and counseling [16–20]. In China, community pharmacies are the most convenient and accessible health care providers (HCPs) for the public due to their geographical proximity to communities and convenient transportation [21]. The focus of community pharmacists in China is gradually shifting from medicine selling to pharmaceutical services such as patient education [22]. They are expected to play an important role in hypertension management, especially in patient education. However, the hypertension management in Chinese communities mainly relies on hospitals and more attention has been paid to the medical staff [23]. But as many scholars emphasize to fully realize the importance of community pharmacists, various circles of society are actively exploring the patterns of hypertension management oriented to community pharmacies, such as the Cooperative Project for Hypertension Management in Pharmacy sponsored by the Chinese Stroke Society and supported by Pfizer [22, 24].

Only 2 studies have reported the positive effect of community pharmacists on hypertension management in China [25, 26]. The number of relevant studies is extremely limited. Also, there is a lack of empirical studies on the situation of hypertension patient education conducted by community pharmacists in China. The complicated and uncertain situation makes it difficult to put forward measures for Chinese community pharmacies to control hypertension. There is an urgent need to carry out research on community pharmacists to provide justifications for the formulation of improvement measures.

In order to fully utilize the role of community pharmacists, it is necessary to understand their current patient education service in hypertension to lay a foundation for improvement measures. This study evaluated the situation of patient education in hypertension conducted in Chinese community pharmacies based on the theory of knowledge, attitude and practice (KAP), and tried to improve their services from various aspects. The results will help to propose possible solutions to the existing problems and provide basis for the formation of supporting policies, thus improving the services and BP control of hypertensive patients to a certain extent. In addition, this study may provide reference for other developing countries, which is conducive to the joint effort to cope with the global burden caused by hypertension.

## Methods

### Study design and participants

In this study, a multi-stage competitive sampling by convenience was used to investigate community pharmacies in the mainland of China, following province / autonomous region / municipality, and prefecture-level city / district, then community pharmacy, and last community pharmacist. Firstly, according to the GDP per capita in 2018, prefecture-level cities in 22 provinces and 5 autonomous regions, and districts in 4 municipalities were evenly divided into three different groups ranging from high, middle and low levels respectively, and one prefecture-level city / district was randomly selected in each level of each province, autonomous region or municipality respectively. Then, one or two community pharmacies that provide hypertension management was selected according to whether the number of permanent residents in the sampled cities / districts was less than 1 million, which was the dividing point of medium and large cities in China. Finally, one community pharmacist was selected from each community pharmacy. According to existing studies, community pharmacists in China mainly consist of a group of young and middle-aged women with a bachelor's degree or less, and have not worked for a long time [27, 28]. Competitive enrollment by convenience was adopted to recruit pharmacists in sampled communities, which means a dynamic adjustment of the number of each group, based on the age, gender, working seniority and other dimensions of pharmacists who have met the inclusion criteria, in order to achieve the expected sampling number as soon as possible and reduce the deviation caused by time. If the community pharmacist refused to participate or didn’t meet the inclusion criteria, the researcher would ask the manager of the community pharmacy to recommend another one, or select another community pharmacy in the prefecture-level city / district.

The inclusion criteria for the sampled community pharmacists were as follows. (1) Full-time community pharmacist working in the sampled community pharmacies, including general pharmaceutical technician who was non licensed pharmacist, but part-time staff or intern was excluded. (2) The working seniority as a full-time community pharmacist was not less than one year. (3) Agreed to participate in the investigation and signed the informed consent.

### Variables and instrument

The theory of KAP is an effective method to help understand the current services of community pharmacists. As one of the commonly used theories in public health, the theory of KAP is easy to be applied and interpreted, which is also characterized by quantifiable data and the generalizability of small samples [29]. It has been widely used around the world to understand the current state of services of health care workers including community pharmacists in a particular area and to lay a foundation for taking measures to optimize it [30–40]. It has been applied to test pharmacists’ knowledge of clinical pharmacy and National Essential Medicine Syste, as well as their attitudes and practice in China [35, 36]. The influencing factors for the services of HCPs may come from many aspects. The theory of KAP holds that knowledge and attitude play a decisive role in practice [37]. In addition, it has been proved that the characteristics of community pharmacists, such as working seniority, will affect the disease management [38].

Based on the theory of KAP, the first draft of the questionnaire was designed from three aspects of knowledge, attitude and practice of community pharmacists on education of hypertensive patients. Delphi method was used to seek consultation from experts to improve the questionnaire, and pre-investigation was carried out to test the reliability and validity of the questionnaire.

The content of the knowledge, practice and attitude sections of the questionnaire was based on the GHEC, guidelines in France and the United States and literature on hypertension prevention and control management [7, 8, 39–41]. The GHEC is jointly compiled by the Chinese Hypertension League, the National Center for Cardiovascular Disease and other organizations. It details patient education in areas such as medical treatment, BP monitoring, dietary habit and exercise method, etc. It is an authoritative material for the practice and necessary knowledge of patient education in hypertension. The questionnaire covers risk factors, complications and hazards of hypertension, treatment of hypertension (medication and healthy lifestyle), self-monitoring of hypertensive patients and hypertension in special populations, belonging to three aspects of knowledge, attitude and practice.

To revise the questionnaire draft, Delphi method was used to consult the expert group, consisting of two senior community pharmacists, two managers of community pharmacies and four professors. There were three rounds of consultation, and electronic consultation forms were sent and recycled in each round. Firstly, the experts were provided with the consultation forms in the first round, which included the questionnaire draft, introduction to the Delphi method, research purposes and related background information for reference. Those forms were recycled to formulate the forms in the second round and seek for consultation again. Then the same method was employed in the third round. Finally, the revision of the questionnaire was completed based on the experts’ feedback.

The final version of questionnaire covered the characteristics, knowledge, attitude and practice of the respondents. The section of characteristics was composed of 6 questions, collecting the demographic characteristics of the respondents and other factors that may affect their KAP, including age, gender, education, working seniority, etc. The section of knowledge was composed of the self-assessment and test of the respondents. Four-point Likert scale was used in the question to collect the result of respondents’ assessment on their knowledge (very good, good, poor, very poor). Their actual level of knowledge was tested with 18 questions, and there was only one correct answer among the four options attached. The section of attitude was based on 4-point Likert scale (totally agree, agree, disagree, totally disagree). It collected the respondents’ attitude towards the role of patient education and themselves by 5 questions. Based on 4-point Likert scale again (always, often, occasionally, never), the section of practice collected the frequency of patient education with 16 questions.

In November 2019, forty five valid questionnaires were collected through convenient sampling in a pre-investigation for community pharmacists, which was conducted in a total of 30 community pharmacies in Nanjing, Xuzhou, Jiangsu Province and Hefei, Anhui Province. In the section of knowledge, the Cronbach’s alpha was 0.783 and the KMO value was 0.729. The approximate chi-square of Bartlett test of sphericity was 445.125, with the degree of freedom being 153, and the *p* value being 0.000. In the section of attitude, the Cronbach’s alpha was 0.915 and the KMO value was 0.834. The approximate chi-square of Bartlett test of sphericity was 392.487, with the degree of freedom being 10, and the *p* value being 0.000. The Cronbach’s alpha was 0.979 and KMO value was 0.928 in the section of practice, in which the approximate chi-square of Bartlett test of sphericity was 2385.574 and the degree of freedom was 120, and the *p* value was 0.000. Therefore the questionnaire was reliable and valid.

### Data collection

In December 2019, two data auditors were responsible for inputting and checking the content of the questionnaire, to ensure that the questionnaire was correctly imported into the online research platform and generated an effective link. In January 2020, five well-trained investigators introduced the purpose, content and significance of the research to the community pharmacists in the sampled community pharmacies through WeChat during non-working hours. With pharmacists’ permission, they asked for the basic information and willingness to participate. Once confirming that the community pharmacists met the inclusion criteria and agreed to participate, the investigator would send the informed consent and the questionnaire link for the respondents to fill in online. They were also responsible for explaining the doubts and recycling the informed consent promptly. Several measures have been taken to ensure the quality of the investigation. Under the investigators’ setting, the questionnaire was allowed to submit until respondents filled in all the items so that there was no missing item in the recycled questionnaires. Each respondent was allowed to fill in one questionnaire on only one electronic device such as a mobile phone or a computer to prevent them from filling in the questionnaire repeatedly. The data auditor can directly review the recycled questionnaires online by logging into the research platform. And questionnaires would be marked as invalid and no longer included in the research, if it was completed less than one minute or the answer to each item tends to be consistent.

### Data analysis

The collected data were imported into SPSS24 software to make descriptive statistics on the characteristics, knowledge, attitude and practice of the respondents. Respondents were grouped according to "whether they had studied the GHEC" and "whether they had received pharmacist training related to hypertension patient education" respectively. The results of the three aspects of knowledge, attitude and practice of hypertension patient education were analyzed by chi-square test or Fisher’s exact test to analyze the differences between the two groups of respondents respectively.

### Ethical consideration

This study was approved by the Ethics Committee of China Pharmaceutical University. All methods were performed in accordance with the relevant guidelines and regulations. Based on the principle of informed consent, all data were collected anonymously after obtaining the permission and informed consent signed by respondents.

## Results

### Participants

One hundred and forty-three questionnaires were collected in this study, among which a total of 108 questionnaires were valid. The efficiency rate was 75.5%. Most of the respondents were women (71.3%), younger than 30 years old (98.1%), and had a bachelor’s degree (95.4%). Only 34.3% of the respondents have learned the GHEC, and 28.7% have received relevant training. (Table 1)

**Table 1:** Characteristics of respondents(*n*=108)

Variable	n (%)
**Age**	
<30	106(98.1)
31-40	1(0.9)
>40	1(0.9)
**Gender**	
Male	31(28.7)
Female	77(71.3)
**Education**	
Below college degree	2(1.9)
College degree	1(0.9)
Bachelor’s degree	103(95.4)
Higher than bachelor’s degree	2(1.9)
**Working seniority as a community pharmacist**	
≤5	105(97.2)
6-10	3(2.8)
>10	0(0)
**Have you studied the Guidelines for Hypertension Education in China**	
Yes	37(34.3)
No	71(65.7)
**Have you received training for pharmacists on patient education in hypertension**	
Yes	31(28.7)
No	77(71.3)

### Knowledge

Generally, respondents didn’t consider that they had grasped the knowledge very well in self-assessment and the results of their test turned out to be average as well, indicating that they have not fully mastered the knowledge on patient education in hypertension. In the section of self-assessment, only 15.7% of the respondents claimed that they had a “very good” command of the knowledge and 43.5% selected “good”, with 30.6% selecting “poor” and even 10.2% selecting “very poor”. Then, the result of test showed that the respondents had a good grasp of the characteristics of hypertension treatment, risk factors, and home self-measurement blood pressure monitors that they would use on a daily basis. About 80% of the respondents knew that hypertensive patients need to be exposed to a long-term treatment (80.6%). Three-quarters of the respondents knew that family history of hypertension is an unchangeable risk factor. More than 70% of the respondents knew the risk factors related to hypertension, and that people with long-term stress, high sodium diet or excessive smoking is susceptible to hypertension (74.1%). More than 60% of the respondents knew the recommended home self-test sphygmomanometer is an upper arm fully-automatic or semi-automatic electronic sphygmomanometer (64.8%). However, the correct response rate of community pharmacists for patient education content with high expertise requirements needs to be improved. The correct response rate for hypertension complications, principles and characteristics of pharmacotherapy, and dietary requirements for nutrients was about 55%. Fewer community pharmacists were aware of the characteristics of home self-measurement of blood pressure (40.7%) compared with the percentage of community pharmacists who knew the recommended type of home blood pressure self-measurement meter (64.8%). The correct rates of questions about the classification and adverse reactions of common antihypertensive drugs were all less than 50%. Only 38.9% of the respondents pointed out the correct answer to the question about the Chinese BMI value of overweight. When it came to the characteristics of hypertension in the elderly, the correct rate turned out to be the lowest, with only 38.0% of the respondents answered correctly. The results of the subgroup analysis showed that whether they had studied the GHEC or whether they had received training related to hypertension patient education differed significantly only in the responses to the individual items, and their overall level of knowledge was generally consistent (Table 2).

**Table 2:** Knowledge of respondents on patient education in hypertension

Item	Correct n(%)	Incorrect n(%)	chi-square test (***p*** value)	
			Have you studied the GHEC	Have you received training for pharmacists on patient education in hypertension
[1] Which population is susceptible to hypertension	80(74.1%)	28(25.9%)	0.075(0.784)	0.218(0.640)
[2] Which one is an unchangeable risk factor for hypertension	81(75.0%)	27(25.0%)	**3.960(0.047)**	0.739(0.390)
[3] According to the standards of Chinese Body Mass Index, people are overweight when their BMI is more than	42(38.9%)	66(61.1%)	0.065(0.799)	0.720(0.396)
[4] Which one is the common complication of hypertension	63(58.3%)	45(41.7%)	1.975(0.160)	0.156(0.692)
[5] Which one is the treatment strategy of hypertension	87(80.6%)	21(19.4%)	0.374(0.541)	0.305(0.581)
[6] Which one is not the principle of anti-hypertensives’ application	60(55.6%)	48(44.4%)	3.289(0.070)	**4.183(0.041)**
[7] Which one is not the characteristic of combined medication for hypertension	63(58.3%)	45(41.7%)	0.339(0.560)	0.001(0.971)
[8] Which one of the descriptions of ACEI^a^ is correct	52(48.1%)	56(51.9%)	0.231(0.631)	0.001(0.975)
[9] Which one of the descriptions of beta blockers is correct	49(45.4%)	59(54.6%)	0.103(0.749)	0.001(0.978)
[10] Which one of the descriptions of CCB^b^ is correct	52(48.1%)	56(51.9%)	1.671(0.196)	0.209(0.647)
[11] Which one of the descriptions of diuretics is correct	49(45.4%)	59(54.6%)	0.812(0.367)	2.827(0.093)
[12] Which one of the descriptions of ARB^c^ is correct	43(39.8%)	65(60.2%)	1.833(0.176)	0.519(0.471)
[13] Which one is the recommended diet for hypertensive patients	60(55.6%)	48(44.4%)	0.995(0.319)	0.579(0.447)
[14] What is the maximum intake of salt for hypertensive patients every day	59(54.6%)	49(45.4%)	0.244(0.621)	0.207(0.649)
[15] Which one is the characteristic of self-measurement of BP at home	44(40.7%)	64(59.3%)	0.632(0.427)	1.053(0.305)
[16] Which one is the recommended home self-test sphygmomanometer	70(64.8%)	38(35.2%)	0.735(0.391)	0.002(0.967)
[17] Which one is the most common cause of hypertension in children	50(46.3%)	58(53.7%)	0.211(0.646)	0.333(0.564)
[18] Which one is not the characteristic of hypertension in the elderly	41(38.0%)	67(62.0%)	**6.188(0.013)**	2.006(0.157)

bold figures: *p*<0.05

^a^*ACEI* Angiotensin Converting Enzyme Inhibitor

^b^*CCB* Calcium Channel Blockers

^c^*ARB* Angiotensin Receptor Blocker

### Attitude

Respondents generally had a positive attitude on the effect of hypertension patient education, but slightly less recognition of their role. About 95% of the respondents affirmed the role of community pharmacists, of which 39.8% "fully agreed". About 99% of the respondents recognized the role of patient education in improving hypertensive patients’ medication compliance, health-related behaviors, and BP control, and helping patients obtain knowledge. Among them, about half completely agreed on these views, and few respondents held a negative attitude. The attitude of hypertension patient education in community pharmacists was almost no difference across groups divided by whether they had studied the GHEC or whether they had received training related to hypertension patient education (Table 3).

**Table 3:** Attitude of respondents towards patient education in hypertension

Item	Completely agree n(%)	Agree n(%)	Disagree n(%)	Completely disagree n(%)	chi-square test (***p*** value)	
					Have you studied the GHEC	Have you received training for pharmacists on patient education in hypertension
[1] Community pharmacists play an important role in the patient education in hypertension	43 (39.8%)	6 (55.6%)	5 (4.6%)	0 (0.0%)	2.860 (0.239)	3.298 (0.192)
[2] Patient education in hypertension can effectively help patients obtain the knowledge of hypertension and medicine	54 (50.0%)	53 (49.1%)	1 (0.9%)	0 (0.0%)	2.316^a^ (0.332)	1.441^a^ (0.569)
[3] Patient education in hypertension can effectively improve medication compliance of patients	56 (51.9%)	51 (47.2%)	1 (0.9%)	0 (0.0%)	2.574^a^ (0.247)	**5.633**^a^ **(0.038)**
[4] Patient education in hypertension can effectively improve patients’ health-related behaviors	52 (48.1%)	56 (51.9%)	0 (0.0%)	0 (0.0%)	1.671 (0.196)	0.209 (0.647)
[5] Patient education in hypertension can help control BP of patients	49 (45.4%)	58 (53.7%)	1 (0.9%)	0 (0.0%)	2.052^a^ (0.383)	1.874^a^ (0.491)

^a^Fisher exact test

bold figures: *p*<0.05

### Practice

Programs related to patient education have been conducted to different degrees. More than 30% of the community pharmacists interviewed implemented them occasionally or never, which is a problem of low frequency of implementation. The program performed most regularly was to “explain the method and key points of self-measurement of BP at home to patients”, which was conducted by nearly 95% of the respondents (always 35.2%; often 30.6%; occasionally 28.7%). The activity performed least regularly was to “explain the damage to the target organ and clinical complications of hypertension to patients”, which was never conducted by 13.9% of the respondents. The situation of other programs were somewhere between the two. It means about 90% of the respondents conducted those activities with different regularities, but only about 30% always did. Whether they had studied the GHEC and whether they had received training related to hypertension patient education showed some differences only in the frequency of implementing patient education on the hazards and complications of hypertension, but the practice of hypertension patient education by community pharmacists was generally consistent across all groups (Table 4).

**Table 4:** Practice of respondents on patient education in hypertension

Item	Always n(%)	Often n(%)	Occasionally n(%)	Never n(%)	chi-square test (***p*** value)	
					Have you studied the GHEC	Have you studied the Education Guide for People with Hypertension
[1] Explain the susceptible population of hypertension	30 (27.8%)	32 (29.6%)	37 (34.3%)	9 (8.3%)	1.473(0.869)	5.100(0.165)
[2] Explain to patients that the earlier stage of hypertension is hard to recognize	35 (32.4%)	36 (33.3%)	29 (26.9%)	8 (7.4%)	4.348(0.266)	6.877(0.076)
[3] Explain the unchangeable risk factors for hypertension to patients	31 (28.7%)	34 (31.5%)	33 (30.6%)	10 (9.3%)	5.614(0.132)	4.693(0.196)
[4] Explain the damage to the target organ and clinical complications of hypertension to patients	30 (27.8%)	39 (36.1%)	24 (22.2%)	15 (13.9%)	1.405(0.704)	5.006(0.171)
[5] Explain the management of hypertension complications to patients	28 (25.9%)	39 (36.1%)	33 (30.6%)	8 (7.4%)	4.684(0.196)	**9.296(0.026)**
[6] Explain the dangerous association of high sodium and low potassium diets with hypertension to patients	38 (35.2%)	35 (32.4%)	26 (24.1%)	9 (8.3%)	7.441(0.059)	4.766(0.190)
[7] Explain the dangerous association of overweight with hypertension to patients	36 (33.3%)	36 (33.3%)	29 (26.9%)	7 (6.5%)	5.411(0.144)	4.162(0.244)
[8] Explain the dangerous association of alcoholism with hypertension to patients	37 (34.3%)	31 (28.7%)	31 (28.7%)	9 (8.3%)	6.055(0.109)	**9.956(0.019)**
[9] Explain the dangerous association of long-term stress with hypertension to patients	33 (30.6%)	36 (33.3%)	31 (28.7%)	8 (7.4%)	2.834(0.418)	6.430(0.092)
[10] Explain the dangerous association of smoking with hypertension to patients	35 (32.4%)	33 (30.6%)	32 (29.6%)	8 (7.4%)	4.116(0.249)	**10.205(0.017)**
[11] Explain the dangerous association of insufficient physical exercise with hypertension to patients	37 (34.3%)	32 (29.6%)	31 (28.7%)	8 (7.4%)	5.191(0.158)	5.754(0.124)
[12] Explain the benefits of a long-term healthy lifestyle for treatment to patients	35 (32.4%)	39 (36.1%)	25 (23.2%)	9 (8.3%)	4.504(0.212)	5.762(0.124)
[13] Explain the basics of medical treatment of hypertension to patients	32 (29.6%)	38 (35.2%)	29 (26.9%)	9 (8.3%)	3.383(0.336)	4.213(0.239)
[14] Explain the characteristic of lifelong treatment for hypertension to patients	29 (26.9%)	43 (39.8%)	29 (26.9%)	7 (6.5%)	3.008(0.390)	5.157(0.161)
[15] Explain the method and key points of self-measurement of BP at home to patients	38 (35.2%)	33 (30.6%)	31 (28.7%)	6 (5.6%)	4.328(0.228)	6.288(0.098)
[16] Explain the specialized treatment of hypertension to special patients such as children and the elderly	30 (27.8%)	34 (31.5%)	36 (33.3%)	8 (7.4%)	4.572(0.206)	5.801(0.122)

bold figures: *p*<0.05

## Discussion

This study explored the situation of patient education in hypertension conducted by community pharmacists by understanding their levels of KAP. It was the first study to explore this issue based on the theory of KAP in China. We found that generally, the respondents were positive about their role in patient education, but they still need to be improved with regard to self-identity, knowledge and practice, which is consistent with the findings reported in other studies conducted in community pharmacists in China that they have positive attitudes but insufficient knowledge about their related duties [27, 42]. This study reflected the current services and revealed the problems of patient education conducted by community pharmacists, which was of vital significance for the later improvement measures.

Respondents generally agreed with the positive role of themselves and patient education, and about half of them fully agreed with this. However, compared to the recognition of the role of patient education, respondents were slightly less likely to recognize the role they play in patient education. This gap may be related to the lack of widespread clinical evidence and authoritative support for hypertension management conducted in Chinese community pharmacies. In China, there is good evidence only indicating that medical staff can achieve clinical effects in the hypertension management [43, 44]. The responsibilities and methods of medical staff to prevent and control hypertension are emphasized in Chinese official guidelines [8, 45, 46]. Those guidelines can be a reference but not a basis directly for community pharmacists, because of the differences in their working environment, responsibilities and limits of authority between community pharmacists and medical staff. It is difficult for community pharmacists to fully identify themselves in the absence of extensive evidence and authoritative support.

To help community pharmacists strengthen their career identity, researches and guidelines are needed to prioritize their responsibilities. It means that more Chinese local researches remain to be done to demonstrate the clinical effects of patient education conducted by community pharmacists. The importance and details of community pharmacists’ responsibilities should be emphasized in guidelines designed for Chinese community pharmacies. Based on international experience and domestic status，new guidelines should be formulated by authoritative institutions or associations as soon as possible. The role of pharmacists in improving patients’ compliance and outcomes and the detailed procedure of patient education have been pointed out by the American Society of Health-system Pharmacists [47]. Considering that drug dispensing is being separated from prescription and the orientation of community pharmacists remains unclarified in China, the new guidelines should accurately define the responsibilities, work processes and evaluation criteria of community pharmacists, and try to refine and standardize their work processes such as establishing health record, health education, follow-up and referral to hospitals.

The low level of knowledge of respondents related to drug therapy, nutrient requirements, and complications with high expertise requirements was associated with insufficient effectiveness of continuing education. The study result showed that only 34.3% of respondents had studied GHEC and 28.7% of them had had relevant training experience and they even still had insufficient knowledge about hypertension, indicating that continuing education needs to be strengthened and improved in China. Continuing education refers to a structured educational activity designed to support the long-term development of pharmacists and/or pharmacy technicians to maintain and enhance their competence [48]. It has been proved effective in obtaining the knowledge of community pharmacists in hypertension management [49]. However, the majority of respondents have not been trained properly on patient education in hypertension, indicating that continuing education has failed to function effectively in China. The continuing professional development (CPD) model in the UK has provided a good example for China, which is equipped with a diversified and strict continuing education system to constantly update the knowledge of pharmacists. The Royal Society of Medicine provides corresponding development planning for the different needs of pharmacy students, new pharmacists and experienced pharmacists. And all the processes are recorded in CPD, which could by checked by the General Pharmaceutical Council irregularly. Those who fails will be disqualified from the Council [50]. In Chinese community pharmacies, community pharmacists consist of licensed pharmacists and general pharmaceutical technicians. Licensed pharmacists are certificated by the provincial medical products administrations for their continuing education, leading to the uneven educational levels between different regions and institutions, and the unsatisfied and diversified needs of licensed pharmacists [51]. General pharmaceutical technicians who receive continuing education more rely on independent participation in training. To improve the efficiency and effectiveness of continuing education in China, a unified certificate authority or standard should be set at the national level with the reference of the CPD model. In addition, education plans should be formulated according to the demands of community pharmacists on basic knowledge and practices at various stages. Under the rigid evaluation of their learning process and results, those who fail the evaluation will be rejected or prohibited from been registered as licensed pharmacists.

Programs on patient education required in the GHEC have not been well conducted by the respondents. As it was found in the research by Sima Garshasbi et al., the respondents were optimistic about patient education but not necessarily would they provide education services all the time [52]. Although all relevant programs have been implemented, there was still a general problem of low frequency of implementation in practice. Some of the items were conducted by only lower than 30% of respondents “always”, such as “explaining the damage to the target organ and clinical complications of hypertension to patients”, “explaining the management of hypertension complications to patients”, and “explaining the susceptible population of hypertension to patients”, etc. These items are all part of the content of health education for patients as stipulated in the GHEC. Only about 30% of the respondents have studied the GHEC and the subgroup analysis showed that whether or not the guideline had been read had little effect on the relevant practice of the community pharmacists interviewed. The dissemination in a limited range and the poor implement of the GHEC also reflected to some extent the weak willingness of respondents to conduct patient education, which hinders the relevant practice.

A study shows that the health care system in the UK would pay for the chronic diseases management provided by community pharmacists, which promotes the wide spread of services [53]. Therefore to promote the implementation of guidelines by community pharmacists, salary incentives can be tried to motivate them. In China, in order to establish a remuneration incentive system related to patient education in hypertension, it is necessary to first improve the continuing education system and formulate unified guidelines to ensure the community pharmacists’ service capacity and standardize their services. On the basis of this, a salary incentive system can be piloted in the chain community pharmacies equipped with the necessary premises, attached facilities and personnel. Those licensed pharmacists who are qualified for patient education are allowed to obtain reasonable and open remuneration according to their performance. Finally, relevant government departments can learn from the experience in the trial processes to improve the salary incentive system and expand the scope of the pilots. More qualified community pharmacists will be allowed to provide fee-based education services for hypertensive patients.

In order to promote the effectiveness of patient education conducted in Chinese community pharmacies, further research can be conducted from the perspective of the factors for services or the needs of hypertensive patients. The distinct economic levels and cultural characteristics in different regions and the low threshold for community pharmacists make the situation more complicated, with diversified factors affecting the service levels of community pharmacists. Analyzing whether and how those factors work is conducive to formulating targeted measures for various groups of community pharmacists. The research on the needs of hypertensive patients could help the skills and knowledge of community pharmacists to satisfy the needs of patients, thus improving the service effect.

This study has limitations in the questionnaire and samples. Since there is no standardized questionnaire for testing the KAP of community pharmacists on patient education in hypertension, this study used a self-designed questionnaire. In order to enhance the scientificity of questionnaire and reflect the reality of community pharmacists’ services effectively, researches based on the theory of KAP and guidelines for hypertension management were referred when the questionnaire was formulated and revised. And Delphi method was adopted to seek consultation from the experts. The result of the pre-investigation indicated that the questionnaire can be used to obtain valuable information about the characteristics, knowledge, attitude and practice of respondents. In terms of the samples, this study focused on the community pharmacies which provide hypertension management, therefore it may not represent all community pharmacists. But it was helpful to obtain more effective information such as the problems existing in the process of patient education.

## Conclusion

The results showed that the service levels of hypertension patient education in Chinese community pharmacies remained to be improved. Community pharmacists held a positive attitude towards the patient education. But there were defects in their knowledge reserve, and at the same time, the GHEC have not been widely implemented in practice. In the future, more Chinese local researches and new guidelines are expected to emphasize the role of community pharmacists and instruct them in patient education. Departments responsible for community pharmacists should improve the continuing education system, and at the national level, certificate those pharmacists in a uniform way and meet their needs at different stages. On this basis, a salary incentive system could be established to try to spur the enthusiasm of community pharmacists.

## Data Availability

The datasets generated and/or analysed during the current study are not publicly available due these data will still be used as auxiliary data in our other studies and we do not wish to publish them publicly but they are available from the corresponding author on reasonable request.

## Abbreviations

BP: Blood pressure
GHEC: Gulidelines for Hyperation Education in China
SBP: Systolic blood pressure
DBP: Diastolic blood pressure
HCPs: Health care providers
KAP: Knowledge, attitude and practice
CPD: Continuing professional development
